# Are artificial intelligence (AI) agents ready for medicine and biomedical research? A narrative review

**DOI:** 10.12701/jyms.2026.43.40

**Published:** 2026-06-17

**Authors:** Sangzin Ahn

**Affiliations:** 1 Department of Pharmacology and Institute of Pharmacogenomics and Precision Medicine, Inje University College of Medicine, Busan, Korea; 2 Cardiovascular and Metabolic Diseases Medical Research Center, Inje University College of Medicine, Busan, Korea

**Keywords:** Artificial intelligence, Automation, Biomedical research, Clinical decision support systems, Patient safety

## Abstract

Artificial intelligence (AI) agents extend large language models from single-turn text generation to systems that pursue goals through planning, retrieval, tool use, code execution, memory, feedback, and role coordination. In medicine and biomedical research, this shift is creating early systems for clinical calculations, risk prediction, oncology decision support, omics analysis, hypothesis development, laboratory automation, and research writing. However, the evidence remains uneven. Clinical examples are the most defensible when agents use validated calculators, curated clinical tools, or guideline-grounded modules under human oversight. Biomedical discovery systems exhibit broader workflow capabilities; however, many claims still rely on preprints, narrow benchmarks, simulated settings, or domain-specific demonstrations. For clinicians and biomedical researchers, the immediate challenge is not to decide whether agents will replace experts but to understand what tasks can be delegated, what evidence is needed, and what human judgment must be preserved. This narrative review explains what makes an AI system agentic, summarizes its representative clinical and discovery applications, and outlines safeguards for evaluation, reproducibility, and oversight. Biomedical readers should expect AI agents to enter medicine and research first as constrained, auditable workflow infrastructures. These infrastructures may reorganize biomedical work; however, accountability should remain with the clinicians and investigators.

## Introduction

Recent attention to “agents” has been driven by the capabilities of large language models (LLMs); however, the underlying idea has predated LLMs by decades. In 1995, Wooldridge and Jennings [1] described intelligent agents as having autonomy, reactivity, proactiveness, and social ability, emphasizing systems that respond to their environment and take goal-directed initiatives. In 1997, Franklin and Graesser [2] sharpened the distinction between ordinary programs and autonomous agents by focusing on situatedness, sensing, action over time, and pursuit of an agenda within an environment. In this tradition, agency refers to more than fluent output. It refers to the capacity to select and execute actions in relation to goals. However, the technical substrate has changed. LLMs now provide a flexible interface for interpreting instructions, decomposing tasks, writing code, calling external tools, retrieving documents, and explaining output. This has made older agent concepts newly relevant to biomedical work.

Unlike a chatbot that answers one turn at a time, a tool-using LLM can call a calculator, database, or code interpreter before answering. An artificial intelligence (AI) agent goes further by maintaining a goal across multiple action-observation cycles, revising its plan, and producing an output that may include a report, recommendation, figure, protocol, or executable artifact. In many implementations, the LLM does not execute actions by itself. It emits a structured tool call or command-like instruction, and an external agent harness executes the action, returns the observation, and inserts the result into the model context for the next step. ReAct provided an early template for this shift by interleaving reasoning with task-specific actions, such as search or environment commands, and then reasoning over the returned observations [3]. Biomedical discovery frameworks now describe agents as systems that connect LLM reasoning to domain databases, computational tools, literature retrieval, planning, and evaluation [4].

This is important because many biomedical tasks are already organized as workflows rather than as isolated questions. Clinical decision support includes patient variables, guidelines, calculators, and evidence. Omics analysis combines data inspection, coding, visualization, and biological interpretation. Laboratory discovery combines hypothesis generation, experimental execution, interpretation of results, and replanning. AI agents are attractive because they can map more naturally onto this workflow structure than single-turn conversational interfaces can.

These systems are no longer technical curiosities but are not yet ready to function as autonomous clinicians or scientists. To date, clinical studies are the strongest when the task is well specified, such as using clinical calculators or structured decision-support tools [5-7]. Biomedical discovery systems have more ambitious workflows, but the evidence base includes a mixture of peer-reviewed studies, preprints, retrospective demonstrations, computational benchmarks, and experimental validation reports [8-17]. For biomedical researchers and clinicians, the practical need is to understand where agentic systems are already useful; where claims remain immature; and what kinds of judgment, documentation, and oversight will become necessary as these tools enter research and care domains. Therefore, this review maps the current landscape of AI agents in medicine and biomedical discovery so that readers can prepare for changes in how biomedical work is organized, delegated, checked, and reported.

## What makes an artificial intelligence system an agent?

**Fig. 1.**
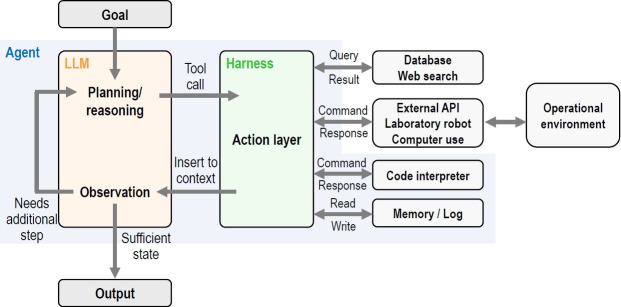
Core artificial intelligence agent loop and components. An artificial intelligence agent contains a large language model (LLM, orange) for cognition and a harness (green) for execution. A user-defined goal enters the LLM, which plans an action and emits a tool call. The harness routes the call to a tool, returns the result as an observation, and the LLM either produces an output or plans the next step. Arrows indicate goal input, tool-call routing, observation return, output generation, iteration, and read or write access to memory and logs. Internal capabilities, including a code interpreter, memory, and log, sit inside the agent. External resources, including databases, application programming interfaces, laboratory robots, and computer-use interfaces, sit outside and let the agent change state in the operational environment. This iterative cycle distinguishes an agentic workflow from a single-turn chatbot response, in the tradition of ReAct [3] and that described by Gao et al. [4].

For this review, an AI agent is defined as a system that pursues a user- or system-defined goal by observing information, choosing actions, using tools, receiving feedback, and updating the next steps. This definition emphasizes function rather than branding. A system should not be called an agent merely because it uses an LLM, provides long answers, or appears conversationally fluent. The defining feature is an action loop of goals, plans, actions, observations, revisions, and outputs (Fig. 1).

In clinical care and biomedical research, autonomy and agency should be graded according to the action space granted by the system. Increased agency can increase automation, speed, and performance because the system can search, calculate, write code, or coordinate steps without waiting for a human prompt at every turn. The same characteristic can propagate errors across a larger workflow, with downstream effects on decisions or actions. Therefore, an agent that autonomously chooses among approved clinical calculators is different from one that writes orders, messages patients, or changes a treatment plan. A wet-lab agent that proposes the next experimental condition is different from one allowed to purchase reagents, operate equipment, or release results without review. The term agent can describe systems with very different benefit-risk profiles.

Biomedical systems can be placed on a spectrum for practical purposes. A chatbot generates a response to a prompt. A retrieval-augmented LLM consults external documents before answering the prompt. A tool-using LLM calls calculators, databases, code tools, or domain models. A workflow agent plans and executes a scoped sequence of actions. A multi-agent or closed-loop system coordinates roles or uses experimental feedback to select the next action. Across this spectrum, the LLM is only one component. The surrounding harness exposes the data sources and tools, stores the state, preserves logs, defines permissions, and returns observations for the next step.

This architecture changes the user’s role. The user no longer simply asks a question and receives an answer but also defines a goal, sets constraints, approves or rejects actions, reviews intermediate work, and determines how the final output enters care or research. For biomedical readers, the key questions are what the system can see, what it can remember, which tools it can access, what actions it can take without approval, and where human responsibility enters the workflow.

## Clinical decision-support agents

Clinical decision support is a natural first domain for biomedical agents, because many clinical tasks already depend on formal tools. Risk calculators, staging systems, diagnostic criteria, guideline pathways, medication rules, and eligibility checks are more suitable for constrained agentic workflows than for open-ended autonomous diagnoses. Clinical calculations are the clearest example. In an evaluation of LLM agents in clinical calculation tasks, Goodell et al. [5] found that task-specific calculator tools reduced incorrect responses from 88% to 16% for LLaMA-based agents and from 64% to 4.8% for Generative Pretrained Transformer (GPT)-based agents, outperforming approaches based on retrieval or code interpretation alone. This result illustrates a core principle. For tasks requiring exact computation or rule application, validated tools should compute, whereas language models should route, explain, and contextualize.

AgentMD extends this concept from individual calculators to a large-scale system to curate and apply clinical risk tools [6]. It extracts risk calculators from the literature and applies them to patient scenarios, emergency department notes, and population-level analyses. The reported performance included 87.6% correctness for computing logic, 89.0% correctness for result interpretation, and 87.7% accuracy on RiskQA, compared with 40.9% for GPT-4. These results suggest that agentic systems can help connect literature-derived risk tools with patient-level information. They also explain why curation and validation are essential. An agent connected to an inaccurate, incomplete, or poorly specified clinical tool can quickly scale errors.

Oncology decision support provides a more complex proof-of-concept. Ferber et al. [7] developed an autonomous AI agent for clinical decision-making in oncology that integrated retrieval, calculators, imaging context, molecular information, and specialist tools. In 20 fictional gastrointestinal oncology cases, the system achieved correct tool use in 56 of 64 required invocations (87.5%), clinical correctness in 223 of 245 response units (91.0%), and higher completeness than GPT-4 alone (87.2% vs. 30.3%). However, potentially harmful responses still occurred in six of the 245 response units, and the citation alignment was 194 of 257 references (75.5%). These numbers support the value of modular agent design in simulated oncology decision support, but they do not establish its prospective clinical utility.

Clinical-agent evidence should, therefore, be read by its distance from the patient-impacting action. Technical demonstrations, retrospective validations, simulated case studies, silent prospective evaluations, pragmatic workflow studies, and interventional deployments are not equivalent. Current evidence supports the use of constrained decision support under human oversight but does not yet justify autonomous clinical action. Before deployment, agent systems require local validation, privacy review, clinician accountability, audit trails, harmful-output monitoring, escalation rules, and clear limits on allowed actions.

## Biomedical discovery and laboratory agents

Research workflows are a natural setting for agents because they involve exploration, iteration, and hypothesis generation. In computational biology, an agent can search the literature, inspect data, write code, run notebooks, debug errors, generate plots, and interpret results. This makes the domain attractive but also difficult to evaluate. The preprint benchmark, BixBench, is a useful counterweight because across 61 realistic bioinformatics scenarios and 205 open-answer questions, the reported open-answer accuracy was approximately 15% to 21% for GPT-4o and Claude 3.5 Sonnet [8]. The practical lesson is that real analysis requires method choice, coding, debugging, assumption checks, figure interpretation, and uncertainty recognition, in addition to biomedical recall.

Several recent systems have illustrated the rapid expansion of biomedical discovery agents. Two preprint system reports illustrate how quickly this space is expanding. Medea focuses on omics analysis and therapeutic discovery with verification and abstention mechanisms [9], and Biomni presents a broader biomedical agent that coordinates tools, retrieval, coding, and domain resources across multiple tasks [10]. As these systems differ in scope, benchmarks, and validation methods, their contributions are best interpreted as architectural and methodological. They show how biomedical resources, tools, and verification steps can be organized into agentic workflows, while leaving open how well such workflows generalize across laboratories, datasets, and clinical settings.

Hypothesis-development systems are among the most visible examples. In a preprint, Gottweis et al. [11] described an AI co-scientist architecture with agents for generation, reflection, ranking, proximity assessment, evolution, and meta-review. Penadés et al. [12] reported a validation companion in which an AI co-scientist mirrored experimental science by recapitulating the mechanism of gene transfer relevant to bacterial evolution, with the experimental biological ground truth reported separately [13]. The value of this example is that it connects agentic reasoning to abductive hypothesis development and prioritization, rather than generic text production. However, it should not be overstated as an independent discovery. Human-defined problems, expert interpretations, and subsequent experimental validations are crucial.

Multi-agent design-and-test systems are closer to experimental biology. The Virtual Lab study used a human-guided multi-agent system to design severe acute respiratory syndrome coronavirus 2 nanobodies and then connected agent outputs to experimental validation [14]. The main contribution was the role-specialized organization of the design, critique, and selection before the experiments, with human guidance and experimental validation still central. This is a plausible near-term model for biomedical research with agents acting as structured participants in supervised workflows.

Closed-loop laboratory agents exhibit the most concrete form of agentic action because they can move from text or code to physical action. Co-scientist, an AI system driven by GPT-4, demonstrated that an LLM-based system could plan and execute chemical research tasks using tools, documentation, code, and robotic laboratory interfaces [15]. ChemCrow demonstrated how expert-designed chemistry tools can augment LLMs for tasks involving molecules, reactions, safety, and search [16]. A preprint on GPT-5-driven cell-free protein synthesis described a closed loop through Ginkgo's cloud lab, where schema-bound plate designs were executed robotically, and fluorescence-derived titer data were fed to the next design round [17]. Such systems matter because agent outputs can influence not only documents but also materials, instruments, and experimental conditions. They also require the strongest constraints, including validated protocols, execution permissions, safety controls, chain-of-custody logging, and human review.

Research automation and publication pipelines add another layer of risk. Lu et al. [18] demonstrated a fully computational AI research agent that performs idea generation, novelty checking, code modification, experimental execution, manuscript writing, and automated reviews without human intervention. One of the three AI-generated submissions received reviewer scores above the acceptance threshold of the International Conference on Learning Representations workshop. This result should be read as a proof-of-concept for computational research automation and not as evidence of generally publishable autonomous science. A preprint analysis, BadScientist, highlights the corresponding risk that agents may generate plausible but unsound papers, particularly when the review is also LLM-supported [19]. A preprint benchmark, OSWorld, extends the same reliability concern to open-ended computer-use tasks, a relevant upstream consideration, because publication-pipeline agents must operate in real software environments rather than answer static questions [20]. Together with the above clinical and discovery examples, these systems demonstrate why the evidence category matters when judging agent readiness (Table 1). For biomedical journals, the use of these systems raises practical questions regarding citation verification, authorship, and peer review integrity. For journal reviews, the more relevant question is not whether text can be classified as AI-generated, but whether the underlying research workflow is transparent, reproducible, attributable, and accountable.

## Evaluation, safety, and reproducibility

Agent evaluation should follow the action space. The more an agent can do, the more evidence and oversight it requires. A literature summary chatbot may require citation checks and expert reviews. A calculator agent requires tool selection and parameter validation. A clinical decision-support agent requires workflow-level testing, harmful-output monitoring, and clinician accountability. A laboratory agent requires biosafety controls, execution approval, and the full provenance of experimental actions.

The final output alone is insufficient. Biomedical agent evaluations should include task success, tool choice, tool parameters, evidence relevance, citation alignment, harmful omissions, calibration, abstention behavior, intermediate artifact reproducibility, auditability, and human workload. The Ferber oncology study is useful because it measured not only clinical correctness, but also tool use, completeness, harmful responses, and citation relevance [7]. Goodell et al. [5] and AgentMD [6] demonstrated the importance of exact tool behavior in clinical calculations and risk prediction. Taken together, these examples support workflow-level evaluations rather than outcome scoring alone.

Multi-agent systems require special transparency. A single-agent system can fail owing to hallucinations, incorrect tool use, or overconfidence. Multi-agent systems add distributed responsibility, hidden intermediate messages, inconsistent shared states, duplicated work, authority confusion, and emergent coordination failures. These risks make transparency a design requirement and not merely a reporting issue, as emphasized in a *Nature Machine Intelligence* editorial on multi-agent systems [21]. In a clinical system with triage, retrieval, calculator, imaging, and summarization agents, the system must preserve the role identity, patient identity, input provenance, action logs, and escalation paths.

Human oversight is not merely a regulatory formality; it is an intellectual aspect of the workflow. Ahn [22] framed this as cognitive load balance. Agents should reduce the extraneous burden and automate well-specified subtasks, while humans retain the germane cognitive work required for judgment, error detection, and scientific skill formation. Lee et al. [23] provided empirical support for this concern. In a survey of 319 knowledge workers and 936 first-hand examples of generative AI use, higher confidence in AI was associated with less self-reported critical thinking, whereas higher task-specific self-confidence was associated with more critical thinking. Collectively, these findings support skill-preserving automation. Agents should offload routine steps without weakening the human capacity to question goals, inspect evidence, and remain accountable for decisions. The practical implication is not to avoid AI assistance but to design agent use to shift human workload from performing every step to actively checking goals, sources, intermediate outputs, and integration into the final product.

Table 2 translates these concerns into a practical checklist. Intended use and permitted actions are central because the risk of an agent depends less on whether it uses an LLM and more on what it is allowed to do, from invoking a calculator to changing code, orders, protocols, or laboratory actions [5-7,15-17]. Tool and data access are included because clinical-calculation and risk-prediction studies show that validated tools and curated source material strongly shape output quality [5,6]. Logging of tool calls, parameters, citations, and retrieved sources addresses provenance, reproducibility, and the risk that plausible outputs may contain unsupported citations or unsound reasoning [7,19,21,24,25]. Realistic validation, abstention, and escalation reflect the gap between benchmark performance and real workflow performance, and the need for systems to defer when uncertainty is high [7-9,22]. Human accountability, privacy and audit requirements, and the distinction between suggestion and action reflect the clinical and institutional responsibilities created when agent outputs enter patient care, research records, unpublished data, or physical laboratory workflows [3,4,7,21-25].

## Future directions

The next stage for biomedical agents should focus less on large demonstrations and more on rigorous, reproducible, and domain-specific validations. Clinical agents require prospective silent trials, pragmatic workflow studies, and interventional studies before they can influence patient care. The evaluations should measure safety, missed findings, clinician workload, time saved, documentation burden, patient-relevant outcomes, and final output correctness.

Biomedical discovery agents require reproducible tool environments, open benchmark tasks, independent expert assessments, and experimental validation to separate the agent contributions from human selection and curation. In omics and computational biology, useful benchmarks should include incomplete data, ambiguous goals, debugging, figure interpretations, and biological plausibility checks. For wet-lab agents, the validation should include safety constraints, protocol fidelity, reagent access limits, and laboratory notebook provenance.

Journals and institutions will also require norms to document AI-supported methods. The main issue is not a categorical distinction between “human work” and “AI work,” because scientific practice is likely to become increasingly hybrid. The more important consideration is whether the workflow is reproducible and checkable, including which model or agent was used, what tools and data it accessed, what code or protocol it produced, what intermediate outputs were retained, and how humans verified the results. This framing is consistent with emerging publisher policies that distinguish routine copy editing from substantive AI use and emphasize documentation, human accountability, and outputs that can be attributed, checked, and verified [24]. Properly documented AI agent workflows could move manuscripts toward two-layer records. Each article would remain optimized for human reading, while the second layer would be agent-native and structured for AI systems to understand, reproduce, and extend the work, rather than for human reading. This distinction sets it apart from conventional supplementary materials, which inherit the same human-optimized, post-hoc structure as the main article. A recent preprint formalized this gap through the Agent-Native Research Artifact framework, noting that narrative publication discards failed experiments, rejected hypotheses, and branching decisions that agents require to avoid repeating dead ends, and omits implementation details that agents need to reproduce the work [25]. Preserving these traces alongside prompts, tool calls, retrieved sources, and intermediate outputs would extend rather than weaken reproducibility, especially when aligned with FAIR (Findable, Accessible, Interoperable, and Reusable) principles [26].

## Conclusion

The important shift is not that LLMs can write longer answers but that model outputs can now be connected to tools, workflows, and physical systems. The same property that allows agents to accelerate biomedical work also allows errors to propagate further before humans notice them. Currently, clinical agents are the most convincing when they use validated tools for narrow tasks under human oversight. Discovery agents are already broader, but their value depends on transparent provenance, realistic validation, and preservation of critical human judgment. For clinicians and researchers, preparation refers to defining action boundaries, inspecting intermediate outputs, and retaining the critical reasoning required to judge hypotheses, analyses, and recommendations. Institutions and journals will need parallel adaptation in documentation and review. The goal is not faster output but skill-preserving automation that offloads routine burden while maintaining human judgment, accountability, and scientific expertise.

## Figures and Tables

**Table 1.** Representative biomedical and scientific artificial intelligence agent systems by evidence category

Domain	Representative example and task	Evidence category	Main lesson and limitation
Clinical calculation and risk prediction	Goodell et al. [5] and AgentMD [6] for calculator and risk-tool workflows	Peer-reviewed scoped and retrospective evaluations	Validated tools can reduce errors, but eligibility, liability, and outcome benefit remain open
Oncology decision support	Ferber et al. [7] with simulated oncology cases	Peer-reviewed simulated cases	Modular design improved completeness, but harmful outputs persisted and prospective utility was not shown
Computational and omics biology	BixBench [8], Medea [9], and Biomni [10] for bioinformatics and omics workflows	Preprint benchmark and early system reports	Agents organize tools, retrieval, code, and verification, but realistic workflows remain difficult
Hypothesis and experimental design	Co-scientist [11], validation companion [12,13], and Virtual Lab [14] for hypothesis and experimental design	Preprints plus peer-reviewed experimental studies	Agents may prioritize hypotheses and designs, but human framing and wet-lab validation remain central
Laboratory automation	Co-scientist [15], ChemCrow [16], and autonomous protein synthesis [17] in chemistry and wet-lab workflows	Peer-reviewed chemistry studies plus wet-lab preprint	Physical action requires protocols, permissions, safety controls, and logs
Research automation	Lu et al. [18] and BadScientist [19] for manuscript and review automation	Peer-reviewed artificial intelligence research plus safety preprint	Automation may accelerate both research and plausible but unsound output

**Table 2.** Practical checklist before using a biomedical artificial intelligence agent

Question	Why it matters
What is the intended use and excluded use?	Prevents uncontrolled expansion from assistance to unsafe autonomy
What actions can the agent take?	Risk depends on whether it can only draft text or can trigger tools, messages, orders, code, or laboratory actions
What tools and data sources can it access?	Tool validity and corpus quality strongly determine output quality
Are tool calls and parameters logged?	Enables error analysis and reproducibility
Are citations and retrieved sources aligned with claims?	Reduces citation hallucination and misinterpretation
Has the agent been validated on realistic cases?	Simple benchmarks may not predict workflow performance
Does it know when to abstain or escalate?	Reduces overconfident errors in uncertain cases
Who reviews the output and remains accountable?	Maintains clinical and scientific responsibility
Are privacy, security, and audit requirements satisfied?	Critical for clinical, omics, and institutional data
Is the output a suggestion or an action?	Distinguishes low-risk assistance from high-risk automation

## References

[1] Wooldridge M, Jennings NR (1995). Intelligent agents: theory and practice. Knowl Eng Rev.

[2] Franklin S, Graesser A, Müller JP, Wooldridge MJ, Jennings NR (1997). Intelligent agents III: agent theories, architectures, and languages. Lecture Notes in Computer Science. Vol 1193.

[3] https://openreview.net/forum?id=WE_vluYUL-X.

[4] Gao S, Fang A, Huang Y, Giunchiglia V, Noori A, Schwarz JR (2024). Empowering biomedical discovery with AI agents. Cell.

[5] Goodell AJ, Chu SN, Rouholiman D, Chu LF (2025). Large language model agents can use tools to perform clinical calculations. NPJ Digit Med.

[6] Jin Q, Wang Z, Yang Y, Zhu Q, Wright D, Huang T (2025). AgentMD: empowering language agents for risk prediction with large-scale clinical tool learning. Nat Commun.

[7] Ferber D, El Nahhas OS, Wölflein G, Wiest IC, Clusmann J, Leßmann ME (2025). Development and validation of an autonomous artificial intelligence agent for clinical decision-making in oncology. Nat Cancer.

[12] Penadés JR, Gottweis J, He L, Patkowski JB, Daryin A, Weng WH (2025). AI mirrors experimental science to uncover a mechanism of gene transfer crucial to bacterial evolution. Cell.

[13] He L, Patkowski JB, Wang J, Miguel-Romero L, Aylett CH, Fillol-Salom A (2025). Chimeric infective particles expand species boundaries in phage-inducible chromosomal island mobilization. Cell.

[14] Swanson K, Wu W, Bulaong NL, Pak JE, Zou J (2025). The Virtual Lab of AI agents designs new SARS-CoV-2 nanobodies. Nature.

[15] Boiko DA, MacKnight R, Kline B, Gomes G (2023). Autonomous chemical research with large language models. Nature.

[16] M Bran A, Cox S, Schilter O, Baldassari C, White AD, Schwaller P (2024). Augmenting large language models with chemistry tools. Nat Mach Intell.

[18] Lu C, Lu C, Lange RT, Yamada Y, Hu S, Foerster J (2026). Towards end-to-end automation of AI research. Nature.

[20] Xie T, Zhang D, Chen J, Li X, Zhao S, Cao R (2024). OSWorld: benchmarking multimodal agents for open-ended tasks in real computer environments. Adv Neural Inf Process Syst.

[21] (2026). Multi-agent AI systems need transparency. Nat Mach Intell.

[22] Ahn S (2025). Preserving critical thinking in the age of large language models: the paradox of cognitive load and efficiency. Korean J Med.

[23] Lee HP, Sarkar A, Tankelevitch L, Drosos I, Rintel S, Banks R (2025). The impact of generative AI on critical thinking: self-reported reductions in cognitive effort and confidence effects from a survey of knowledge workers. In: Proceedings of the 2025 CHI Conference on Human Factors in Computing Systems.

[24] https://www.nature.com/nature/editorial-policies/ai.

[26] Wilkinson MD, Dumontier M, Aalbersberg IJ, Appleton G, Axton M, Baak A (2016). The FAIR Guiding Principles for scientific data management and stewardship. Sci Data.

